# Nanoscale Control of Aggregation-Induced Emission and Second Harmonic Generation in a Dicyano Distyrylbenzene-Appended Polymer Using Optical Trapping

**DOI:** 10.1021/acs.jpcb.5c04092

**Published:** 2025-07-29

**Authors:** Shun-Fa Wang, Kuan-Chih Tseng, Mahiro Nakabayashi, Shotaro Hayashi, Fumitaka Ishiwari, Teruki Sugiyama

**Affiliations:** † Department of Applied Science, 63285 National Taitung University, Taitung 950309, Taiwan; ‡ Department of Applied Chemistry and Center for Emergent Functional Matter Science, 34914 National Yang Ming Chiao Tung University, Hsinchu 300093, Taiwan; § School of Engineering Science and Engineering, 47743 Kochi University of Technology, Kochi 782-8502, Japan; ∥ FOREST Center Research Institute, Kochi University of Technology, Kochi 782-8502, Japan; ⊥ Department of Applied Chemistry, Tokyo Metropolitan University, Tokyo 192-0397, Japan; # Division of Materials Science, Graduate School of Science and Technology, Nara Institute of Science and Technology, Ikoma, Nara 630-0192, Japan

## Abstract

We demonstrate nanoscale control over aggregation-induced emission (AIE) and second harmonic generation (SHG) by using optical trapping to manipulate a cyano-substituted distyrylbenzene (CDSB)-substituted polymer. The precise manipulation afforded by a tightly focused laser beam induces the formation of micrometer-sized polymer aggregates, demonstrating control over structure at the microscale. These aggregates exhibit yellow fluorescence with a prominent emissive species at 577 nm. Notably, further laser irradiation generates distinct aggregates characterized by blue fluorescence and strong SHG emission, indicating the formation of noncentrosymmetric microstructures with enhanced nonlinear optical properties. We elucidate the dynamics and mechanisms governing these disparate aggregation behaviors, highlighting the potential of optical trapping to control both AIE and SHG for the rational design of advanced functional microstructured materials and photonic devices, such as optical switches or frequency converters.

## Graphical abstract

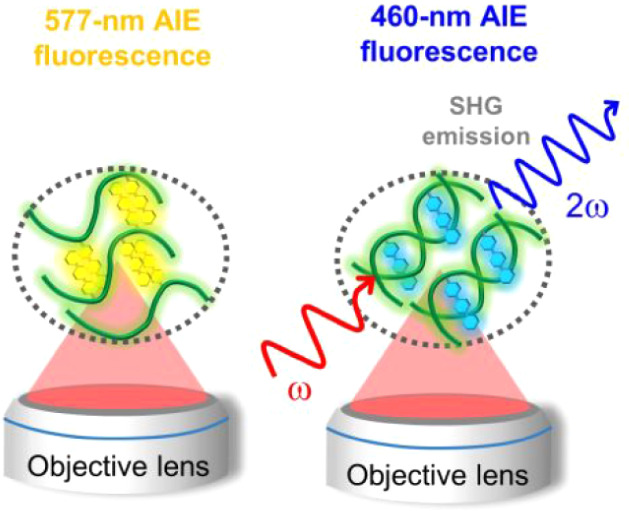

## 1 Introduction

Aggregation-induced emission (AIE) provides a powerful strategy for designing functional materials where light emission is regulated by molecular aggregation, effectively overcoming the limitations of traditional dyes that suffer from concentration quenching.^1^ The ability to harness AIE has led to advancements in various fields, including organic light-emitting devices,^2,3^ bioimaging,^4–6^ biosensing,^7,8^ and others.^9–14^ Nevertheless, in most cases, the AIE phenomenon is activated by altering the solution’s pH or changing the solvent from a good to a poor one. While these methods induce aggregation and restrict intramolecular rotations (particularly those responsible for nonradiative decay in AIE molecules), precise control over AIE at the nanoscale—essential for fabricating next-generation optoelectronic and sensing devices—remains a key bottleneck.

In contrast to these traditional approaches, optical trapping offers a novel and intriguing physical method for AIE activation, utilizing optical forces to control the aggregation of AIE-active molecules.^15^ Optical trapping (or optical tweezers) is a noncontact, three-dimensional manipulation technique that uses a highly focused laser beam to precisely manipulate nano- and micrometer-scale objects.^16,17^ This powerful technique has become an indispensable tool in interdisciplinary research, enabling the control of micro/nanoscale organization and dynamics in physics, chemistry, and biology.^18–22^ The fundamental principle of optical trapping relies on gradient forces exerted by the focused laser beam on targets at the focal point, where the electric field is strongest, leading to their accumulation and a significant increase in local concentration. Our research group has been exploring the potential of optical trapping to induce and control AIE. We recently reported a unique phenomenon, “optical trapping-induced AIE,” observed in a tetraphenylethene (TPE) derivative^15^ and a TPE-functionalized polymer,^23^ both well-known AIE-active compounds. In those studies, optical trapping induced aggregate formation and activated their specific AIE by applying optical forces to the forming aggregate, a departure from traditional AIE activation through chemical condition changes. Notably, optical trapping-induced AIE was achieved by enhancing molecular association within the aggregate and suppressing nonradiative decay pathways (e.g., twisting of the central ethylene and rotation of the phenyl rings in TPE). This capability allowed for reversible switching of AIE fluorescence in single aggregates by adjusting the trapping laser power. Furthermore, continued laser irradiation of the aggregate generated a highly concentrated domain exhibiting two distinct AIE fluorescence emissions at different wavelengths. This unique optical response indicated the presence of discrete nanoenvironments surrounding the polymer-linked TPE molecules.

This study investigates optical trapping-induced AIE in a polymer system with a different AIE mechanism, employing a polymer appended with a dicyano distyrylbenzene (CDSB) moiety.^24,25^ Given the distinct fluorescence properties and AIE mechanism of CDSB compared to TPE,^26,27^ we aim to compare the results from this CDSB-appended polymer with our previous TPE-based systems. This comparative approach will provide deeper insights into the underlying mechanisms of optical trapping-induced AIE and further demonstrate the technique’s versatility. We utilize steady-state fluorescence and fluorescence lifetime measurements to analyze the aggregation dynamics and resultant aggregates formed by optical trapping. This approach enables us to probe the temporal evolution of AIE and investigate the molecular aggregation, conformations, and intermolecular arrangements at the micro/nanoscale within the aggregates. Ultimately, we seek to elucidate how optical trapping influences the polymer chains and appended CDSB moieties to induce AIE and control the aggregates’ optical properties.

## 2 Experimental Section

### 2.1 Sample Preparation

A dye-appended polymer (poly-CDSB_0.005_), constituted of a poly­(dimethylaminoethyl acrylate) (PDMAEA) and (*Z*)-3-{4-[(*Z*)-2-cyano-2-phenyl-1-ethenyl]­phenyl}-2-phenyl-2-propenenitrile, also referred to as cyano-substituted distyrylbenzene (CDSB), was employed as the sample for optical trapping experiments. The synthetic route for the sample is depicted in Figure S1. The poly-CDSB_0.005_ polymer had an estimated molecular weight of approximately 10.13 kg/mol, with a polydispersity index of 9.04. The sample solution was prepared at a concentration of 10 μM by dissolving 2.5 mg of poly-CDSB_0.005_ into D_2_O (Sigma-Aldrich, 99.9%). During the trapping experiments, D_2_O was used as a solvent to mitigate local temperature elevation resulting from laser heating.^19^ The sample solution, contained within a light-protected glass bottle, was stirred overnight at 650 rpm using a magnetic stirring bar. A custom-made glass chamber with an approximate volume of 1.0 cm^3^ was cleaned with an alkaline detergent solution (Hellma, Hellmanex III; concentration: 10% v/v) in an ultrasonic cleaner (DELTA, D80) for 10 min. The glass chamber was subsequently cleaned twice with deionized water in the ultrasonic cleaner immediately prior to the optical trapping experiment. A 10 μL aliquot of the sample solution was deposited into the chamber, forming a thin solution film with a thickness of 100 μm on the highly hydrophilic glass surface. The chamber was then covered with a glass slide and positioned on the stage of an inverted microscope (Olympus, IX71) for subsequent trapping experiments.

### 2.2 Optical Setup

Figure S2 provides a schematic representation of the optical trapping setup. A continuous-wave 1064 nm Nd^3+^:YVO_4_ laser (Coherent, Matrix 1064-10-CW), serving as the trapping light source, had its radial diameter expanded by two convex lenses (focal lengths *f* = 100 and 150 mm) to match the pupil diameter of the objective lens. The laser beam was then tightly focused at the air/solution interface using a high-numerical-aperture oil-immersion objective lens (100×, N.A. 1.4) on the microscope. The laser power after the objective lens was adjusted to 700 mW by rotating the optical axis of a half-wave plate positioned in front of a polarizing beam splitter. A 405 nm laser (Spectra-Physics, Excelsior 405), used as the excitation light source, was directed into the microscope, with its power after the objective lens set to 2 μW. Trapping behavior was monitored using a charge-coupled device (CCD) camera (Watec, WAT-231S2) in both transmission and fluorescence imaging modes. Under 405 nm illumination, the fluorescence emission at the laser focus passed through a confocal pinhole (diameter: 200 μm) within a confocal scanning system (Olympus, FV-300) and was detected by a spectrometer consisting of a polychromator (Princeton Instruments, Acton Spectra Pro 2300i) and a CCD camera (Princeton Instruments, PIXIS 400). The accumulation time for each fluorescence spectrum was 1 s. A custom-built microspectroscopic system was utilized to measure the fluorescence lifetime of the aggregates produced by optical trapping. Details of the optical setup are provided in Figure S3. All optical experiments were conducted at room temperature (25 °C).

## 3 Results and Discussion

### 3.1 Optical Trapping-Induced AIE and Spectral Dynamics

We employed optical trapping to manipulate a CDSB-appended polymer, poly­(dimethylaminoethyl acrylate) (PDMAEA) with 0.5 mol % CDSB (poly-CDSB_0.005_), and investigated its AIE behavior at the air/solution interface. The chemical structure of poly-CDSB_0.005_ is shown in Figure S4. Figure 1 depicts the aggregation formation at the laser focal spot and the corresponding fluorescence images. Before starting the laser irradiation, the solution was clear, with no visible aggregates (Figure 1a, panel (i)), and negligible fluorescence emission was observed at this stage (Figure 1b, panel (i)). Upon laser irradiation, a submicrometer-sized aggregate, designated as the yellow-emitting aggregate (YEA), was formed at the laser focal spot within 3 min (Figure 1a, panel (ii)), and showed faint fluorescence emission (Figure 1b, panel (ii)). Continued irradiation led to YEA growth, as evidenced by darkening in the transmission image, resulting from reduced light penetration (Figure 1a, panel (iii)). This growth was accompanied by a rise in fluorescence intensity (Figure 1b, panel (iii)). After 15 min of irradiation, the YEA reached approximately 2.5 μm in diameter and exhibited strong fluorescence (Figure 1a, panel (iv)). The fluorescence emission of the YEA could be clearly observed (Figure 1b, panel (iv)). These observations confirmed the successful optical trapping of poly-CDSB_0.005_ and its AIE activation.

**Figure 1:**
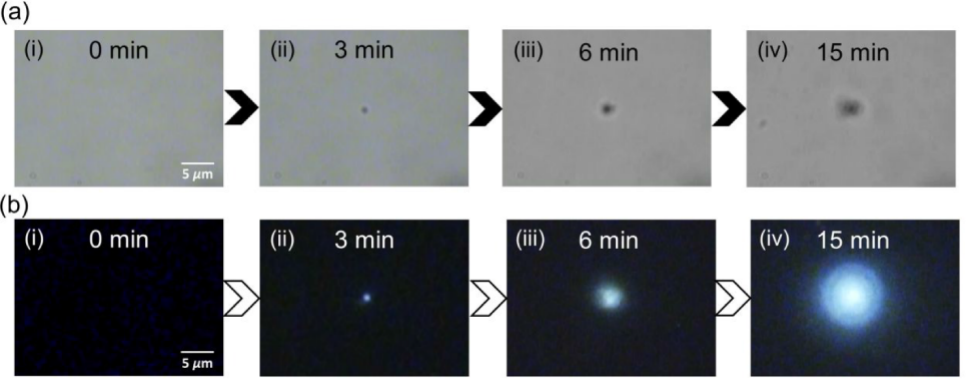
Optical trapping and microscale aggregate formation. (a) Transmission images showing the time evolution of aggregate growth at the laser focal spot, demonstrating microscale manipulation by optical trapping. (b) Corresponding fluorescence images under 405 nm illumination, illustrating the activation of AIE during aggregate formation.

The temporal evolution of the YEA fluorescence spectrum was then investigated during 15 min of laser irradiation using confocal microspectroscopy (Figure 2). Initially, no fluorescence emission was observed in the 420–700 nm range. After 3 min, YEA was formed, and its AIE emerged, with a peak at 575 nm and a shoulder around 470 nm. The fluorescence intensity of YEA increased significantly with irradiation time (Figure 2a). Figure 2b displays the normalized fluorescence spectra of the YEA during laser irradiation (3–15 min), providing a basis for analyzing the temporal evolution of both fluorescence intensity and peak wavelength, as summarized in Figure 2c. Notably, Figure 2c reveals a nearly monotonic increase in peak intensity throughout the irradiation period, accompanied by a gradual blue shift from 575 to 567 nm. The asymmetric shape of the spectrum and the spectral shift suggest the presence of multiple fluorescent species within the YEA. In fact, spectroscopic analysis of the poly-CDSB_0.005_ solution revealed that the CDSB moiety itself exhibits weak fluorescence emission at 460 nm, as shown in Figure S5. This intrinsic fluorescence, however, was not readily detectable under the initial microscopic observation conditions (Figure 2a).

**Figure 2:**
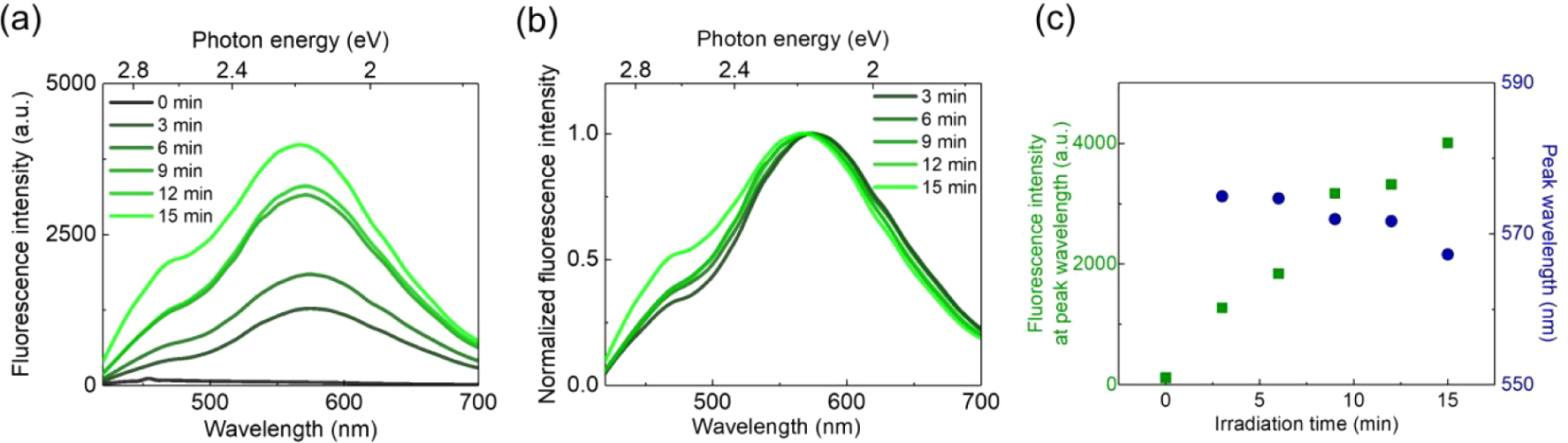
(a) Time evolution of YEA fluorescence spectra by optical trapping. (b) Normalized fluorescence spectra of YEA during optical trapping. (c) Fluorescence intensity and peak wavelength as a function of irradiation time.

To determine the number of fluorescent species within the YEA, a Gaussian curve fitting method was employed. Initially, two Gaussian fitting curves were used, as the exact number of species was unknown. The fitting parameters, including peak wavelength and full width at half-maximum (fwhm), were determined for the known fluorescent species in order to obtain a calculated fitting curve for the unknown species. The detailed fitting procedure and results are presented in Figure S6. Based on the results mentioned above, it was reasonable to assume that the CDSB moiety constituted one of the fluorescent species in the YEA. However, the fitting with two curves was unsuccessful, as the calculated spectrum showed a significant shift in peak position with irradiation time. This result strongly suggested the presence of at least one additional unknown fluorescent species within YEA. To identify the unknown species, the emission properties of PDMAEA polymers alone were analyzed under the same optical trapping conditions. Surprisingly, the trapped PDMAEA aggregate exhibited a broad fluorescence emission with a peak at 504 nm under 405 nm excitation (Figure S7), despite the lack of a large π-conjugated system in the polymer. This unexpected finding suggests that optical trapping activates AIE in the PDMAEA polymer. While further investigation of this intriguing phenomenon is warranted, this study focused on the AIE behavior of the CDSB-appended polymer under optical trapping conditions. With the identification of two fluorescent species emitting at 460 and 504 nm, it became possible to explore the third fluorescent species using fitting methods.

A three-Gaussian curve fitting (Figure 3a) revealed a distinct emission peak centered at 577 nm, which remained temporally stable over 15 min (Figure 3b). The peak wavelength consistently remained at 577 nm, and the reliability of these fitting results is supported by the coefficient of determination (*R* ^2^) exceeding 0.997 for all fitted spectra. Having established the presence of this third fluorescent species, which emits at 577 nm, it is necessary to define the abbreviated terms for each species. The three fluorescent species within YEA were designated as follows: the green-emitting species (GES) from the PDMAEA aggregate with emission at 504 nm, the blue-emitting species (BES) from the CDSB moiety with emission at 460 nm, and the yellow-emitting species (YES) with emission at 577 nm. Figure 3c shows the fluorescence intensity of the three distinct emissive species, revealing the evolving nanoscale composition of the aggregate over 15 min of laser irradiation. The fluorescence intensity of all three species increased throughout the irradiation period. Notably, the fluorescence intensity derived from BES initially exceeded that from GES. In contrast, after 9 min of irradiation, the intensity from GES surpassed that from BES, indicating a more pronounced increase in GES emission. This suggests that as optical trapping progresses, the AIE intensity from the PDMAEA aggregate becomes dominant over the AIE emission from the CDSB moiety. Additionally, it became clear that BES and GES contributed to the shoulder at shorter wavelengths. On the other hand, the fluorescence intensity derived from YES also increased with laser irradiation time, and it remained the primary emitting species from the YEA throughout the 15 min irradiation period. The origin and nature of YES will be discussed in detail in Section 3.3. This fitting analysis reveals the compositional changes of the three fluorescent species within the optically trapped YEA.

**Figure 3:**
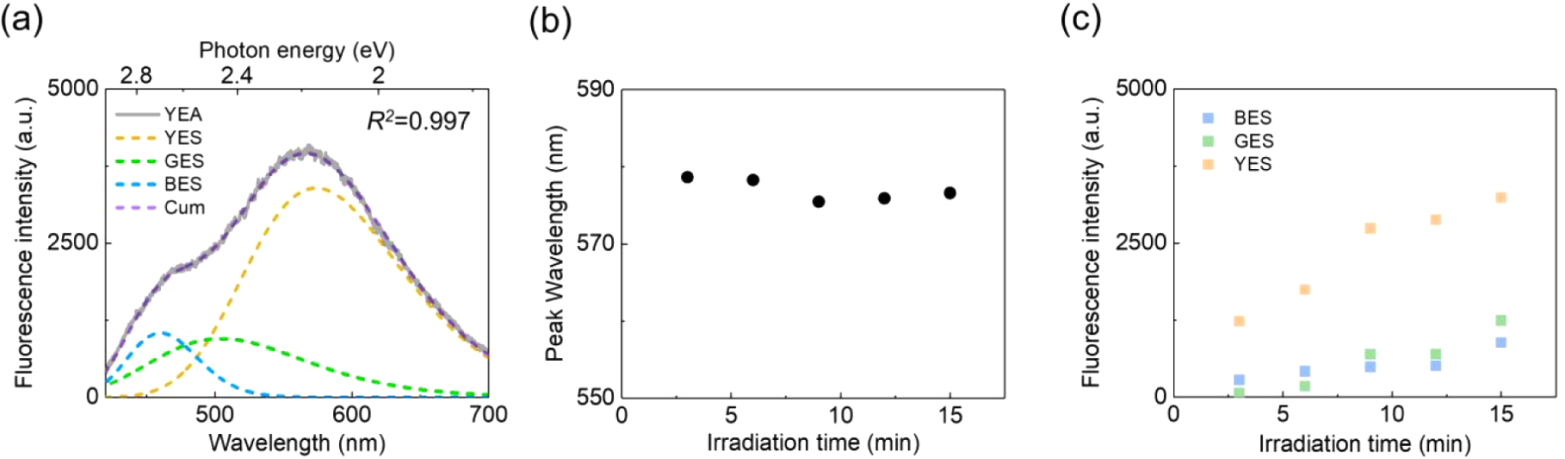
(a) Representative Gaussian curve fitting of the fluorescence spectrum, showing three independent emission bands. The gray solid line represents the YEA emission at 15 min; yellow, green, blue, and purple dashed lines represent YES, GES, BES, and the cumulative fit (Cum), respectively. (b) Peak wavelength of YES as a function of irradiation time. (c) Temporal changes in the peak fluorescence intensity of YES, GES, and BES with irradiation time.

### 3.2 Microscale Control of Aggregate Morphology and Nanoscale Composition

In most optical trapping experiments, similar aggregation behavior and fluorescence changes were observed as described in Section 3.1. However, in approximately 25% of trials, a distinct aggregation behavior was observed, characterized by a sudden shift in the YEA fluorescence to blue. Figure 4 shows fluorescence images of this particular behavior after 5 min of wide-area excitation illumination. During irradiation, the aggregate formed at the focal spot sometimes split. Some smaller aggregates migrated away and are subsequently retrapped at the edge of the initial aggregate, resulting in an irregular shape. A new, circular aggregate with blue fluorescence then emerged outside the focal spot (Figure 4a, panel (i)). This blue aggregate was optically trapped at the focal spot, displacing the YEA to the surrounding solution (Figure 4a, panel (ii)). The blue aggregate remained stably trapped for over 100 s, though the fluorescence weakened slightly (Figure 4a, panel (iii)). The corresponding transmission image shows a relatively dark aggregate (∼2 μm diameter) due to decreased light transmission (Figure 4b). This aggregate is designated as the blue-emitting aggregate (BEA) to distinguish it from YEA.

**Figure 4:**
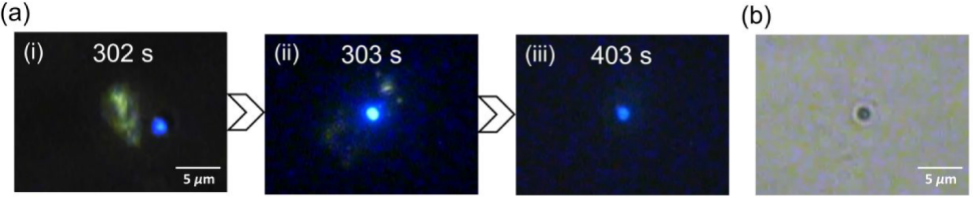
(a) Fluorescence images showing representative trapping behavior of BEA at 5 min irradiation, obtained under 405 nm wide-field excitation. (b) Transmission image of BEA trapped at the focal spot.

The BEA formation mechanism is of great interest. Notably, the BEA always appeared outside the focal spot. This can be explained by the formation of a high-concentration domain (HCD) around the focal spot. The HCD generation via optical trapping has been previously reported in many systems.^23,28,29^ Initially, optical trapping increases the local polymer concentration, leading to aggregate formation (Figure 1a, panel (iii)). Continuous irradiation generates HCD in the vicinity of the focal spot, likely due to gradient optical forces extending beyond the focal point, capturing surrounding polymers. The HCD reduces the difference in refractive index between the trapped aggregate and the surrounding solution, thereby weakening the trapping efficiency and inducing aggregate splitting. This suggests that the seemingly single aggregate in Figure 1a may be an assembly of smaller aggregates, highlighting the complex hierarchical organization that can be achieved and probed at the microscale using optical trapping. The BEA is likely formed spontaneously within the HCD or from YEA that has migrated out of the focal spot and transformed within the HCD. The BEA composition was then investigated. Comparison of normalized fluorescence spectra of BEA and YEA (Figure 5a) reveals a large peak wavelength difference of ∼115 nm. Notably, the BEA spectrum overlaps significantly with that of the mother solution (Figure S8). Gaussian fitting revealed BES as the major fluorescent species in the BEA, with minor contributions from GES and YES (Figure S9).

**Figure 5:**
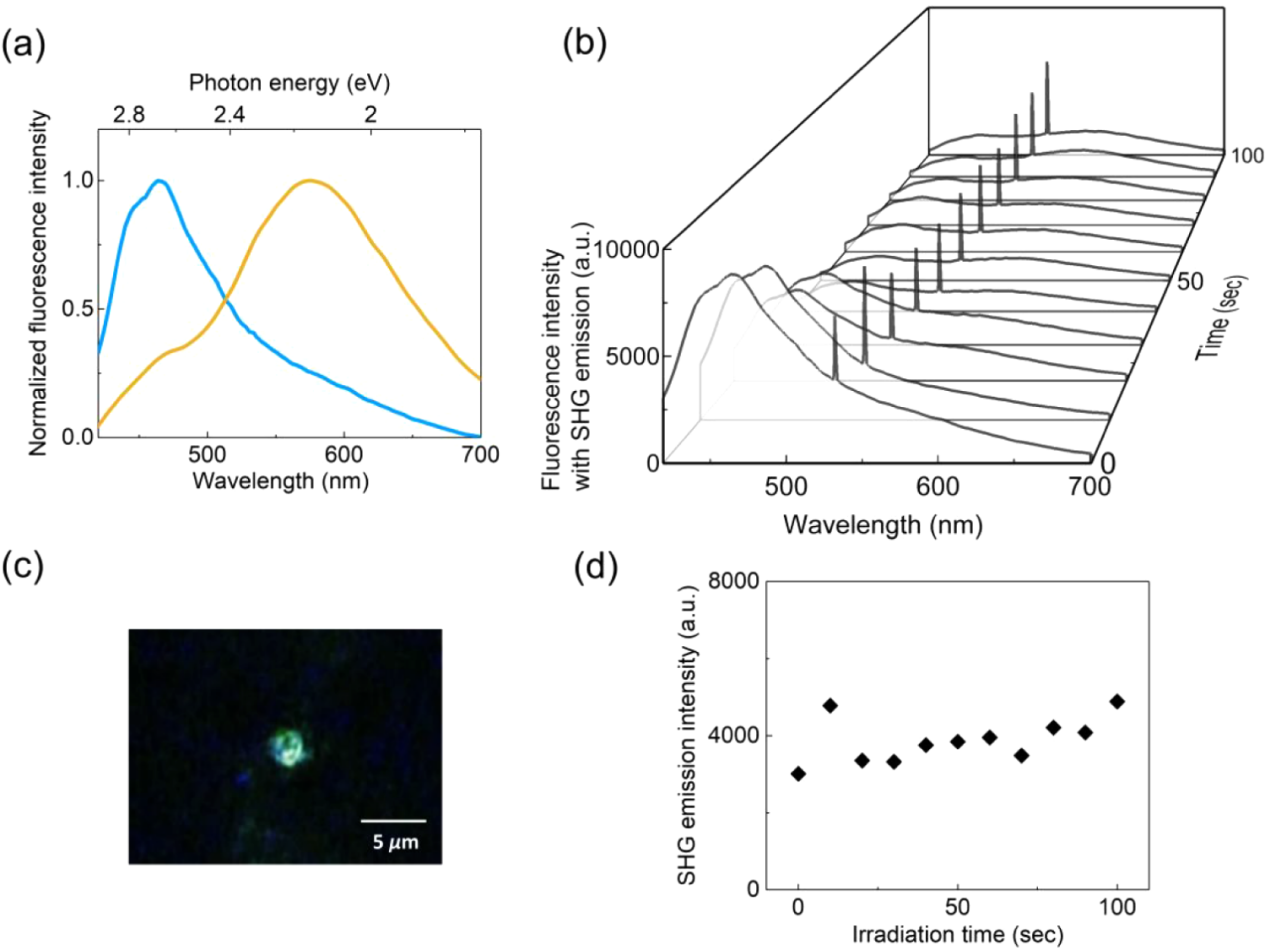
(a) Normalized fluorescence spectra of BEA (blue line) and YEA (yellow line). (b) Time evolution of fluorescence spectra of BEA during optical trapping. The spectra include a sharp SHG emission peak at 532 nm. (c) Microscopic image of the SHG emission. No excitation laser irradiation at 405 nm throughout the image measurement. (d) SHG emission intensity as a function of irradiation time.

### 3.3 Fluorescence Lifetime Analysis of Aggregates

As demonstrated in the previous sections, both BEA and YEA contain three fluorescent species: BES, YES, and GES, with BES and YES being the major components in BEA and YEA, respectively. To gain structural insights into these species, we conducted fluorescence lifetime measurements on BEA and YEA, which were prepared using the same procedures described earlier. Table 1 summarizes the fluorescence lifetimes, and the fluorescence decay curves are illustrated in Figure S10.

**Table 1:** Fluorescence Lifetimes of BEA and YEA Produced by Optical Trapping^a^

	τ_1_ (ns)	*A*_1_ (%)	τ_2_ (ns)	*A*_2_ (%)	τ_3_ (ns)	*A*_3_ (%)
BEA	1.1	93	3.9	7	-	-
YEA	1.0	57	4.4	34	17.1	9

a BEA lifetimes were measured with a 430 nm bandpass filter to exclude YES emission, revealing two components (BES and GES). YEA measurements showed a lower contribution from the YES component due to simultaneous excitation of the aggregate and surrounding solution containing BES and GES.

We first discuss the fluorescence lifetime measurement of BEA, performed by blocking YES fluorescence with a bandpass optical filter (420–440 nm transmission). Two fluorescent components with τ values of 1.1 and 3.9 ns were detected. Given its dominant intensity in the decay curve, the short fluorescence lifetime component (1.1 ns) is assigned to BES, in agreement with the findings from the previous section. Consequently, the other component (3.9 ns) is attributed to GES. Next, we measured the fluorescence lifetime of YEA without the bandpass filter. In addition to the lifetimes above, an additional component with a τ value of 17.1 ns was observed and assigned to YES. We compared the fluorescence lifetimes of BES and YES with previously reported data.^24^ The BES lifetime (1.1 ns) closely matches that of isolated CDSB molecules dispersed in a poly­(methyl methacrylate) film (1.35 ns). This suggests that BES originates from individual CDSB molecules enveloped by polymer chains. Conversely, the YES lifetime (17.1 ns) is an order of magnitude longer than that of BES. This indicates the formation of a stable dimer-like aggregate of CDSB, characterized by a red-shifted emission. The GES lifetime (3.9 ns), representing the emissive component of the PDMAEA polymer, is first attained in this work.

### 3.4 Second Harmonic Generation (SHG) Emission from BEA

A particularly interesting phenomenon, providing insight into the BEA composition, was observed from the BEA trapped at the focal spot. Figure 5b shows the fluorescence spectral evolution over 100 s, with *t* = 0 corresponding to BEA being captured (Figure 4a, panel (ii)). The *t* = 0 spectrum shows a strong, sharp emission at 532 nm, exactly half the trapping laser wavelength, in addition to the 460 nm emission characteristic of YEA. Intriguingly, the 532 nm peak persisted even after the excitation laser was ceased, and the emission was visually observed during the experiment as shown in Figure 5c. These results indicate that the 532 nm peak can be attributed to second-harmonic generation (SHG). In this nonlinear optical effect, two incident photons are combined to double the incident light frequency.^30,31^ The SHG emission intensity as a function of irradiation time is shown in Figure 5d. The SHG emission remained stable for over 100 s before weakening or disappearing, eventually transitioning from BEA to YEA. This SHG generation suggests a noncentrosymmetric arrangement of poly-CDSB_0.005_ within BEA.

Here, we discuss the SHG observed from BEA. Notably, strong SHG light is consistently detected from BEA, but rarely from YEA. Generally, SHG emission, a nonlinear optical process, occurs when a strong laser irradiates a material lacking inversion symmetry (noncentrosymmetric structure).^30,31^ In such structures, the induced polarization is not linear with the applied electric field, leading to the generation of light at the second harmonic frequency. The SHG observed from BEA suggests that poly-CDSB_0.005_ adopts this noncentrosymmetric arrangement within BES. Notably, SHG emission was also generated from the PDMAEA polymer alone under optical trapping, albeit with a low probability of 20% (two out of ten trapped aggregates exhibited SHG). This experimental result provides crucial evidence that the noncentrosymmetric arrangement upon aggregation may be an intrinsic property of PDMAEA. When CDSB is appended to PDMAEA, the intermolecular association of the polymer is enhanced, further reinforcing the polymer structure in the aggregate. Additionally, the appended CDSB increases the polymer’s polarizability. Consequently, the SHG effect is significantly improved in BEA. On the other hand, in YEA, the close association of paired CDSB moieties hinders the formation of the noncentrosymmetric arrangement of the polymer, significantly suppressing SHG generation. Therefore, SHG emission is hardly observed in YEA, which has a dimer-like arrangement of CDSB. This study represents the first example in our research group of inducing SHG in an aggregated polymer using optical trapping, demonstrating a novel pathway for creating microscale nonlinear optical materials with tunable properties.

### 3.5 Proposed Mechanism for Distinct Aggregate Formation by Optical Trapping and Implications for Nanoscale Organization

This study reveals a significant finding: the spatiotemporal dynamics of AIE-active YES formation induced by optical trapping. This exhibits a remarkable redshift of approximately 117 nm (0.55 eV) compared to the solution. To understand this unique fluorescence emission, we consider the internal structure of YES in relation to known literature on polymorphic crystals of CDSB.^24^ CDSB molecules in chloroform solution emit fluorescence at 447 nm. Spontaneous nucleation under ambient conditions leads to the formation of three crystal polymorphs of CDSB with interplanar stacking (H-aggregates), exhibiting fluorescence maxima at 480, 514, and 531 nm. The relationship between emission wavelength, molecular dihedral twist, and intermolecular arrangement has been thoroughly discussed in previous work. Notably, the largest Stokes shift (84 nm, 0.44 eV) is associated with a crystal structure characterized by reduced intramolecular torsion and enhanced side-by-side packing. These specific nanoscale arrangements are believed to be responsible for the observed spectral shift. Building on this, we propose that the even larger Stokes shift (0.55 eV) observed in our optically trapped YES arises from a highly ordered planar structure. In this structure, CDSB exhibits minimal intramolecular torsion and a high degree of orbital overlap between paired CDSB molecules, effectively forming a thermodynamically stable dimer with ideal face-to-face stacking. This precise control over nanoscale molecular arrangement leads to the observed longer-wavelength emission.

We now turn to the mechanism by which optical trapping spatially separates the formation of YEA at the focal position and BEA outside the focal spot, as well as the underlying mechanism of optical trapping-induced AIE. Figure 6 illustrates this mechanism. Initially, poly-CDSB_0.005_ in solution exhibits weak fluorescence emission at 460 nm, primarily from the CDSB moiety (Figure 6a). Upon laser irradiation, gradient forces (fN to pN) and scattering forces drive the assembly of polymers and their clusters toward the focal spot, resulting in a rapid increase in local concentration. The contribution of scattering force to this concentration increase is a distinctive feature of our air–solution interface experiments. This increased concentration at the focal point promotes intermolecular association between the polymers (Figure 6b), with CDSB moieties associating strongly via hydrophobic interactions. The seemingly disproportionate influence of the low CDSB concentration (0.5%) is consistent with our previous observations in TA-TPE-appended polymers. Consequently, CDSB dimers surrounded by polymers are generated in the aggregate, leading to dimer emission at 577 nm (Figure 6c). Notably, the aggregate initially trapped at the focal spot invariably exhibits this dimer emission, indicating that optical trapping effectively drives the formation of these specific nanoscale CDSB dimer structures and the resulting YEA.

**Figure 6:**
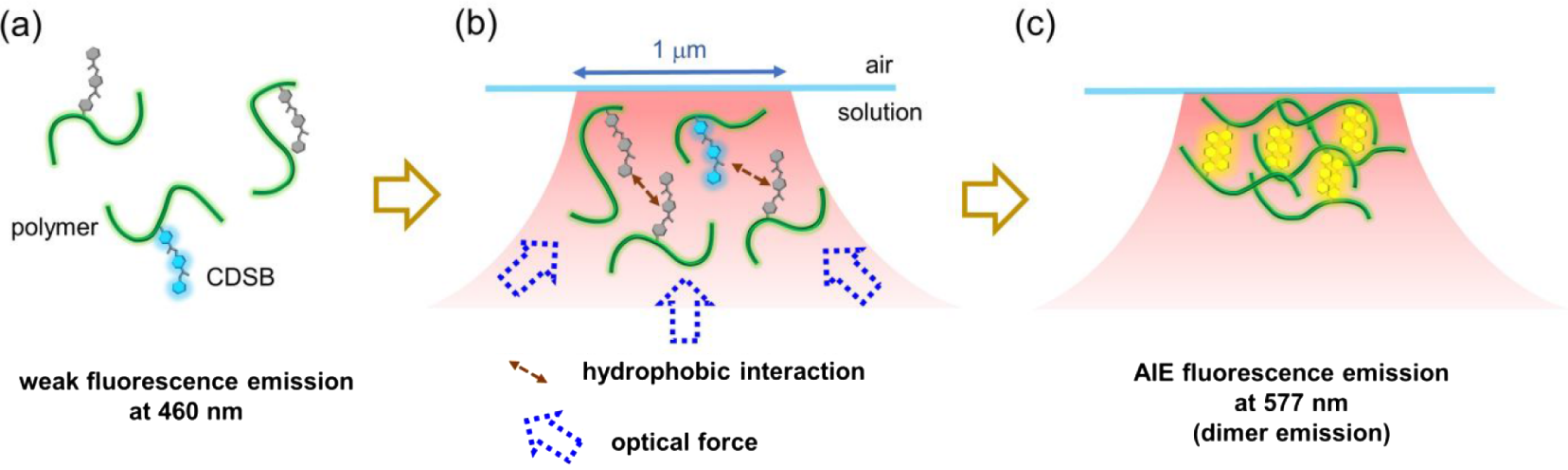
Proposed mechanism for aggregation and fluorescence emissions of poly-CDSB_0.005_ by optical trapping.

Conversely, continued laser irradiation of the trapped aggregate further increases the concentration at the focal spot, and this increased concentration extends outward, forming a high-concentration domain (HCD) near the focal point. Within this HCD, polymer clusters and small aggregates can grow slowly with reduced optical trapping force. Under these conditions, hydrophobic interactions between CDSB moieties are less dominant. Consequently, CDSB moieties are individually dispersed within the polymer, activating AIE fluorescence emission at 460 nm, and BEA is generated. The transition from YEA to BEA within the HCD is likely driven by the destabilization of the CDSB dimer structure due to weakened optical forces, which increases the molecular freedom of the CDSB units. This increased freedom allows for their reclustering into a more stable, BEA structure within the polymer matrix. Thus, optical trapping induces YEA formation through direct optical forces while simultaneously creating an HCD conducive to BEA formation. The distinct AIE fluorescence emissions provide insight into the structural evolution of the aggregates and the precise control over nanoscale molecular association achieved by optical trapping.

## 4 Conclusion

In conclusion, we elucidated the spatiotemporal dynamics of aggregation-induced emission (AIE) in a poly­(dimethylaminoethyl acrylate) (PDMAEA) polymer appended with a cyano-substituted distyrylbenzene (CDSB) molecule (poly-CDSB_0.005_) under optical trapping. Notably, optical trapping induced the formation of distinct fluorescent species—BES, GES, and YES—each characterized by unique emission wavelengths, revealing a high degree of control over the aggregate’s nanoscale composition. Optical trapping of poly-CDSB_0.005_ results in the formation of yellow-emitting aggregates (YEA) at the laser focal spot. AIE activation in YEA is attributed to enhance molecular association, specifically hydrophobic interactions between CDSB moieties, leading to the formation of thermodynamically stable CDSB dimers. These dimers exhibit a red-shifted emission at 577 nm, a significant 117 nm shift compared to the solution state. The AIE fluorescence of YEA thus primarily originates from this CDSB dimer emission, a species rarely observed in CDSB alone. The polymer chains, under the influence of optical trapping forces, effectively promote the formation of these emissive dimers. While dimer emission is proposed as the primary AIE mechanism of YEA, the potential contribution of CDSB excimers warrants further investigation.

Conversely, blue-emitting aggregates (BEA) were generated in the high-concentration domain (HCD) surrounding the focal spot. In BEA, CDSB moieties are individually dispersed within the polymer matrix, resulting in AIE fluorescence at 460 nm from the emissive BES component. Consequently, optical trapping enables the spatial separation of YEA and BEA, each displaying distinct AIE characteristics dictated by the differential molecular associations between the polymer chains and CDSB moieties. Intriguingly, we observed 504 nm fluorescence from the PDMAEA polymer itself (GES), suggesting “clustering-triggered emission” from close associations between subunit groups. It is also possible that the observed green-emitting species (GES) is related to clusteroluminescence, as recently reported in studies on nonconjugated aliphatic polymers containing abundant heteroatoms,^32,33^ given that optical trapping may promote the polymer’s own clustering and induce this emission, which offers a promising avenue for further exploration concerning the precise mechanism.

A key finding is the selective generation of second harmonic generation (SHG) in BEA, but not (or rarely) in YEA. This selectivity is governed by the intermolecular associations of CDSB and the polymer within the aggregates. Strong SHG emission is associated with individually dispersed CDSB in a noncentrosymmetric arrangement, whereas CDSB dimer formation inhibits this noncentrosymmetric structure. Remarkably, this SHG behavior is highly sensitive to the small proportion (0.5%) of CDSB within the polymer. This observation presents a novel strategy for designing functional microscale photonic devices with nanoscale control over nonlinear optical properties, such as optical switches or frequency converters.

This work demonstrates a novel approach to precisely control and investigate both nonlinear optical phenomena and AIE at the microscale using optical trapping of AIE-active molecule-appended polymers, offering significant potential for the development of advanced functional microstructured materials and photonic devices.

## Supplementary Material



## Notes

Detailed information on materials, general characterization methods (NMR, UV–vis, SEC, MS), and synthesis procedure of poly-CDSB_0.005_ (PDF)
