# Supplementary material for: Nanoscale Control of Aggregation-Induced Emission and Second Harmonic Generation in a Dicyano Distyrylbenzene-Appended Polymer Using Optical Trapping
Source: J Phys Chem B. 2025 Jul 29;129(31):8065–72. doi: 10.1021/acs.jpcb.5c04092 (PMC12337084; doi:10.1021/acs.jpcb.5c04092)
Supplement: Supplementary file 1 [file jp5c04092_si_001.pdf]

Supporting Information

Nanoscale Control of Aggregation-Induced Emission and Second Harmonic Generation in a Dicyano Distyrylbenzene-Appended Polymer Using Optical Trapping

Shun-Fa Wang[1,2*], *Kuan-Chih Tseng*[2], *Mahiro Nakabayashi*[3], *Shotaro Hayashi*[3,4], *Fumitaka Ishiwari*[5], *and Teruki Sugiyama*[2,6*]

[1]Department of Applied Science, National Taitung University, Taitung 950309, Taiwan, [2]Department of Applied Chemistry and Center for Emergent Functional Matter Science, National Yang Ming Chiao Tung University, Hsinchu 300093, Taiwan, [3]School of Engineering Science and Engineering, Kochi University of Technology, Kochi 782-8502, Japan, [4]FOREST Center Research Institute, Kochi University of Technology, Kochi 782-8502, Japan, [5]Department of Applied Chemistry, Tokyo Metropolitan University, Tokyo 192-0397, Japan, [6]Division of Materials Science, Graduate School of Science and Technology, Nara Institute of Science and Technology, Ikoma, Nara 630-0192, Japan

Table of Contents for Supporting Information

Figure S1. Synthetic scheme for poly-CDSB$_{0.005}$). S3

Figure S2. Schematic diagram of the optical trapping system with confocal microspectroscopy. S3

Figure S3. Schematic diagram of the custom-built microspectroscopic system for fluorescence lifetime measurements. S4

Figure S4. Chemical structure of the poly-CDSB$_{0.005}$ polymer. S4

Figure S5. Fluorescence spectrum of the poly-CDSB$_{0.005}$ sample solution measured by a commercial fluorescence spectrometer. S5

Figure S6. (a) Fluorescence spectrum of the yellow-emitting aggregate (YEA) fitted with two Gaussian curves, and (b) temporal change of the peak wavelength of the yellow-emitting species (YES). S5

Figure S7. Fluorescence spectrum of a single PDMAEA aggregate formed by optical trapping....................S6

Figure S8. Normalized fluorescence spectra of the blue-emitting aggregate (BEA) and the bulk solution of poly-CDSB$_{0.005}$, with SHG emission removed. S6

Figure S9. Fluorescence spectrum of the blue-emitting aggregate (BEA) resolved into three Gaussian curves, with SHG emission removed. S7

Figure S10. Fluorescence decay curves of the blue-fluorescent aggregate (BEA) and the yellow-fluorescent aggregate (YEA). S8

Additional Experimental Details.

Detailed information on materials, general characterization methods (NMR, UV-vis, SEC, MS), and synthesis procedure of poly-CDSB$_{0.005}$.

Figure S11. ^{1}H NMR spectrum of compound 3 in $CDCl_3$. S9

Figure S12. ^{13}C NMR spectrum of compound 3 in $CDCl_3$. S10

Figure S13. Absorption spectra of compound 3 and poly-CDSB$_{0.005}$. S10

Figure S14. SEC trace of poly-CDSB$_{0.005}$. S11

Reference S11

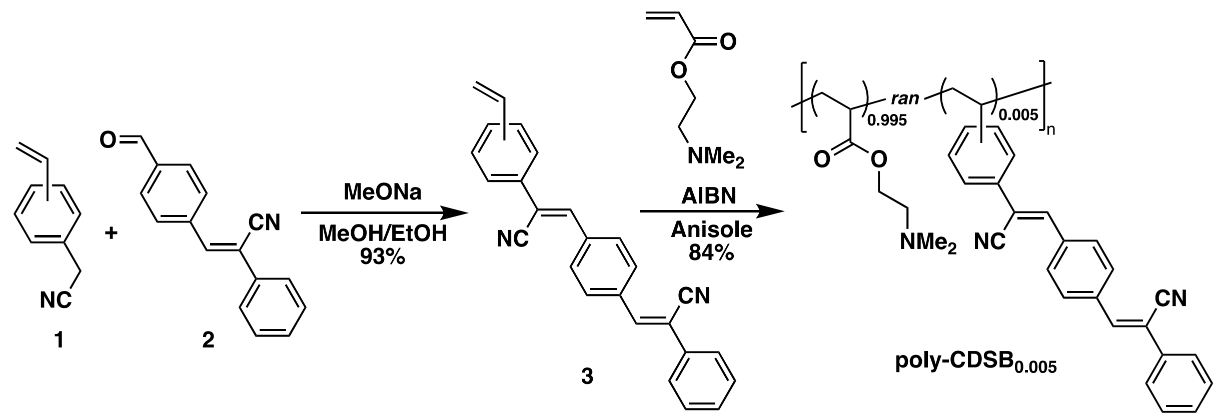

Figure S1. Synthetic scheme of poly-CDSB$_{0.005}$.

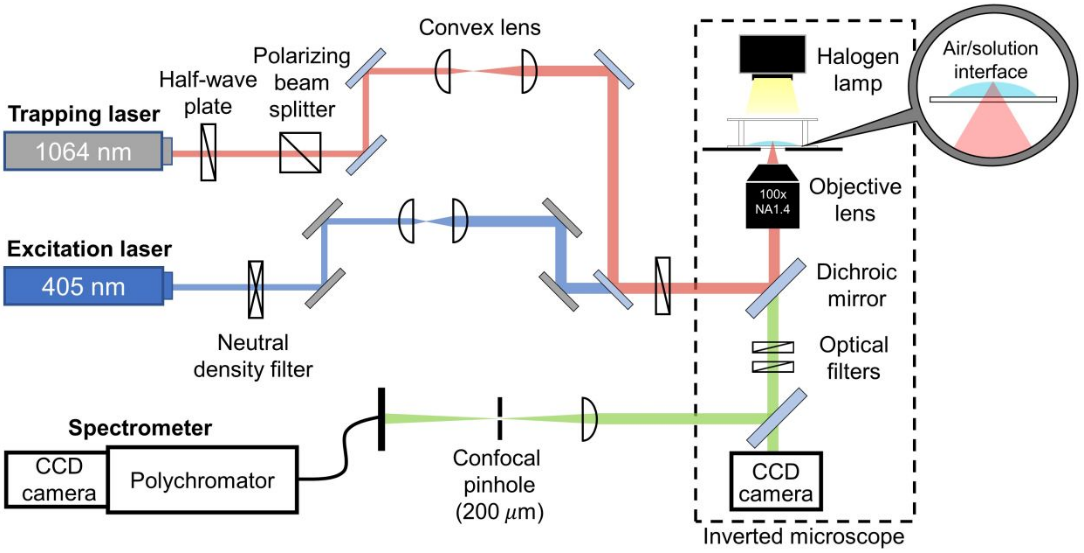

Figure S2. Schematic diagram of optical trapping with the confocal microspectroscopic system.

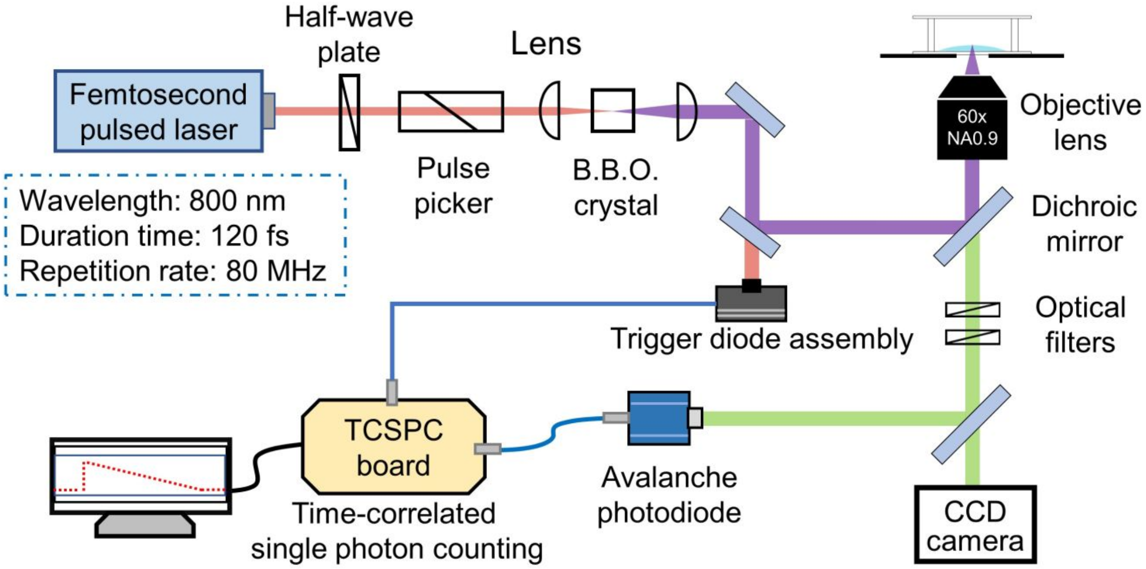

Figure S3. Schematic representation of the custom-built microspectroscopic system employed for fluorescence lifetime measurements. The excitation source utilized was a femtosecond pulsed laser (Spectra-Physics, Tsunami) operating at 800 nm with a repetition rate of 80 MHz. The wavelength and repetition rate of the pulsed laser were modulated to 400 nm and 8 MHz, respectively, using a pulse picker (ConOptics, Model 350-160) and a BBO crystal. This modulation resulted in a temporal separation of 125 ns between consecutive pulses. The modulated laser pulses were subsequently focused onto the target aggregate via a high numerical-aperture objective lens. To eliminate backscattered light, a 405-nm long-pass optical filter was employed. Time-correlated single photon counting (TCSPC) (PicoQuant, PicoHarp 300) was employed to determine the fluorescence lifetime, with the emitted photons detected by an avalanche photodiode (Micro Photon Devices, PDM Series). The instrument response function (IRF) of the TCSPC system, characterized by analyzing the scattering light from a glass substrate, was estimated to be about 0.25 ns.

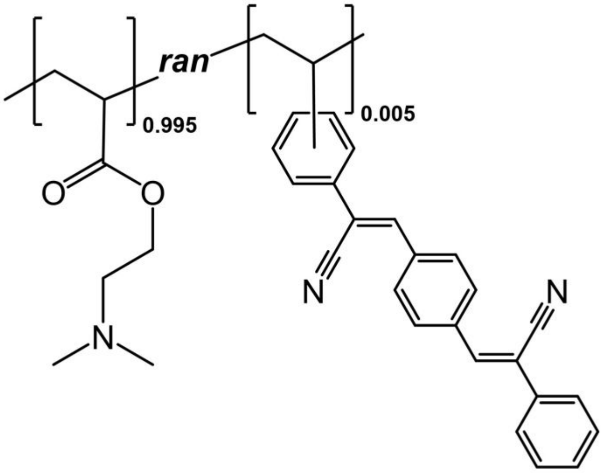

Figure S4. Chemical structure of poly-$CDSB_{0.005}$.

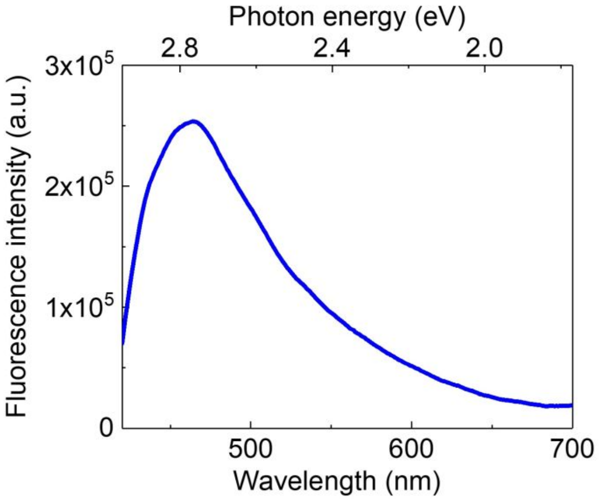

Figure S5. Fluorescence spectrum of the poly-$CDSB_{0.005}$ sample solution obtained using a commercial fluorescence spectrometer (Horiba, FluoroMax plus).

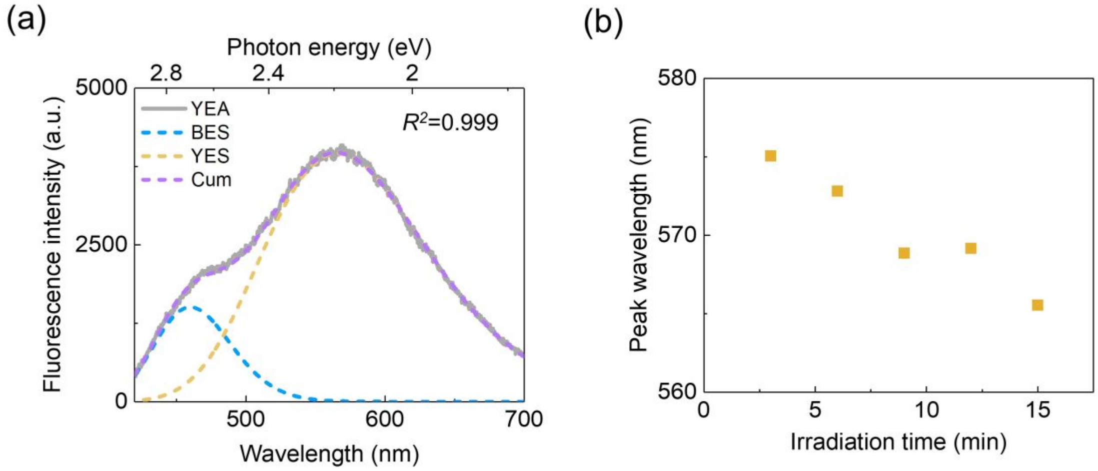

Figure S6. (a) Fluorescence spectrum of YEA resolved into two Gaussian fitting curves. The solid gray line represents the fluorescence band of YEA at 15 min, while yellow, blue, and purple dashed lines represent YES, BES, and the cumulative fit (Cum), respectively. (b) Temporal change of the peak wavelength of YES, determined by the fitting method. A spectrum shift in nearly 10 nm, larger than the spectral resolution of our instrument, was observed.

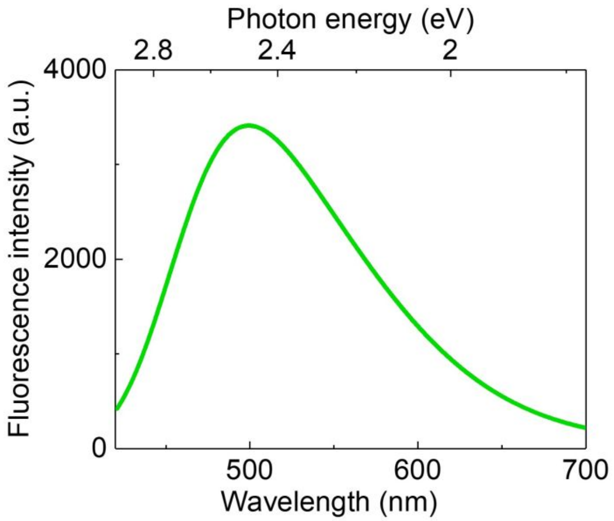

Figure S7. Fluorescence spectrum of a single PDMAEA aggregate formed by optical trapping. The trapping conditions and solution concentration of the PDMAEA polymers were the same as those of the poly-CDSB$_{0.005}$ trapping experiment in the main text.

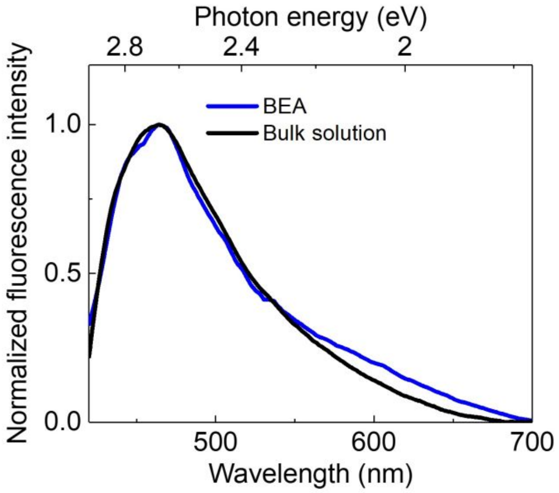

Figure S8. Normalized fluorescence spectra of BEA and the bulk solution of poly-CDSB$_{0.005}$. The SHG emission at 532 nm from BEA was removed.

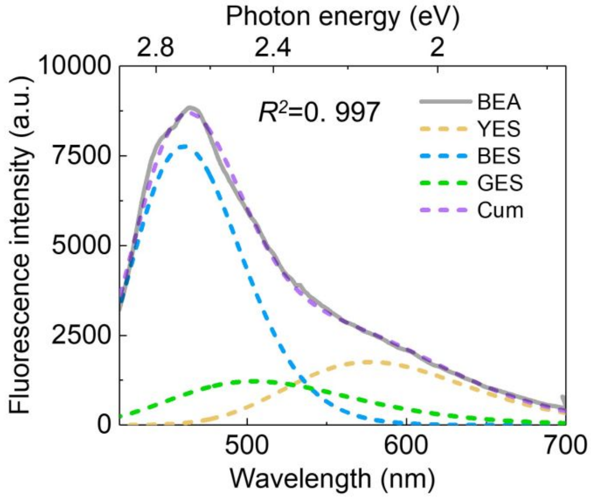

Figure S9. Fluorescence spectrum of the blue-emitting aggregate (BEA) resolved into three Gaussian curves, with SHG emission removed.

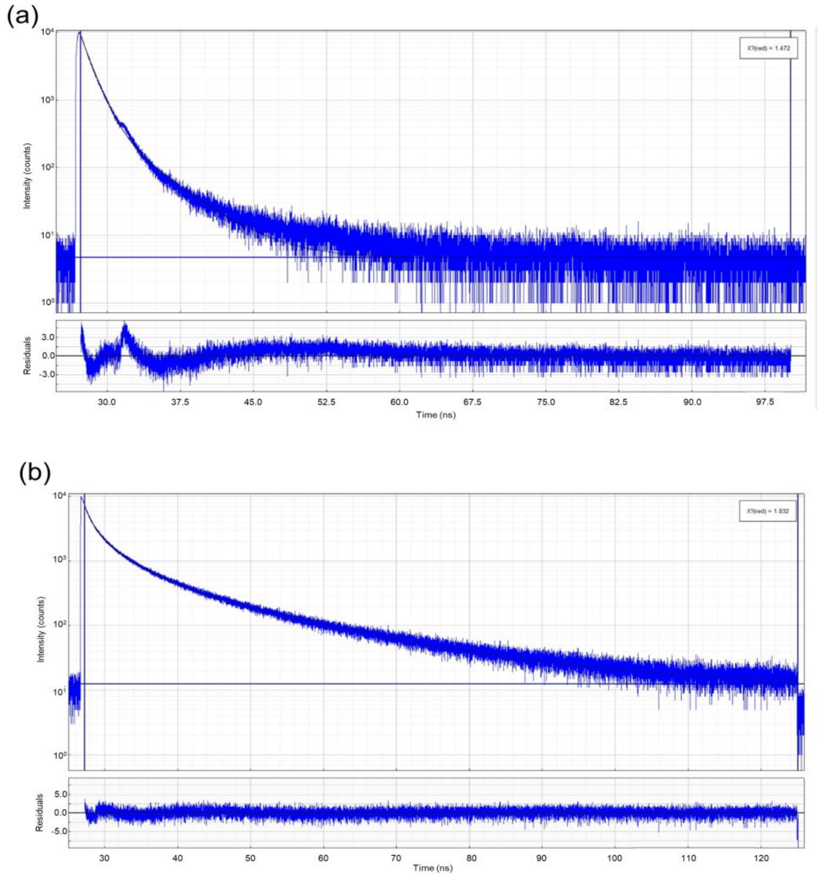

Figure S10. Fluorescence decay curves of (a) BEA and (b) YEA.

Additional Experimental Details.

Materials.

Vinylphenylacetonitrile (**1** in Figure S8, *meta*-, *para*-mixture, Aldrich) and 1 M sodium methoxide in methanol (Wako) were used as received. 2-(dimethylamino)ethyl acrylate (TCI) was passed through an Al_2O_3 short column to remove radical stabilizer MEHQ just before use. Compound **2** in Figure S8 was synthesized according to the previous report[S1] and unambiguously characterized by NMR spectroscopy and atmospheric pressure chemical ionization time-of-flight (APCI-TOF) mass spectrometry.

General.

NMR spectra were recorded at 25 ˚C on a Bruker model AVANCE-400, a JEOL ECZ400S spectrometer (400.0 MHz for 1H and 100.6 MHz for ^{13}C) or a JEOL JNM-ECS400 (400.0 MHz for 1H and 100.6 MHz for 13C). Chemical shifts (δ) were determined with respect to residual non-deuterated solvent for 1H (CDCl3: 1H(δ) = 7.26 ppm) and residual solvent for ^{13}C ($CDCl_3$: $^{13}C(\delta)$ = 78.0 ppm, CD_2Cl_2: $^{13}C(\delta)$ = 53.2 ppm). The absolute values of the coupling constants are given in Hertz (Hz), regardless of their signs. Multiplicities are abbreviated as singlet (s), doublet (d), multiplet (m), and broad (br). UV-vis absorption spectra were recorded at 25 ˚C using a JASCO model V-730 or V-650 UV-vis spectrometer. Analytical size-exclusion chromatography (SEC) was performed at 40 ˚C regulated by column oven (CO-4060) on a JASCO ChromNAV GPC system equipped with an UV detector (UV-4075) and a refraction index (RI) detector (RI-4030), using dimethylformamide (DMF) containing LiBr (0.01 M) as an eluent at a flow rate of 0.50 mL/min (PU-4180) on a column (TSKgel SperAWM-H, TOSOH). The molecular weight calibration curve was obtained using standard polystyrenes (TSK standard polystyrene, Tosoh). APCI-TOF MS spectrum was performed on a Bruker compact QTOF mass spectrometer in acetonitrile (Kanto Chemical Co.). Calibration was performed with Tuning Mix (Agilent Technologies) in the range of 100 *m*/*z* to 1000 *m*/*z*.

Synthesis of poly-CDSB$_{0.005}$.

An anisole solution (1.06 mL) of a mixture of **3** (12.6 mg, 0.021 mmol), 2-(dimethylamino)ethyl acrylate (1.06 mL, 6.99 mmol), and AIBN (11.5 mg, 0.07 mmol) was degassed by freeze-pump-thaw cycles (three times) and purged with argon. The mixture was stirred at 65 ˚C for 24 h and then allowed to cool to 25 ˚C. The reaction mixture was poured into *n*-hexane, and the precipitate formed was collected by decantation and dried under reduced pressure to afford poly-CDSB$_{0.005}$ (84% yield) as a yellow paste (0.84 mg, corresponding to 5.87 mmol of the monomer unit). Assuming that the molar absorption coefficient (ε) of compound **3** and the obtained polymer at λ_{max} = ca. 350 nm are identical, the composition ratio of the CDSB

was consistent with the feed ratio of $x = 0.005$. Although close to the detection limit of ^{1}H NMR spectroscopy, the integration ratio of ^{1}H NMR signals supports this composition ratio.

^{1}H NMR (400 MHz, $CDCl_3$): δ (ppm) 8.0-7.4 (br, 0.075H), 4.13 (br, 2H), 2.53 (br, 2H), 2.33 (br, 1H), 2.25 (s, 6H), 1.90 (br, 1H), 1.65 (br, 1H). $M_n = 1.54$ kg mol^{-1}; $M_w/M_n = 2.81$. Analytical data (UV absorption spectra, ^{1}H NMR spectra, and SEC trace) of poly-CDSB$_{0.005}$ are shown in Figures S13, S11, and S14, respectively.

Analytical Data

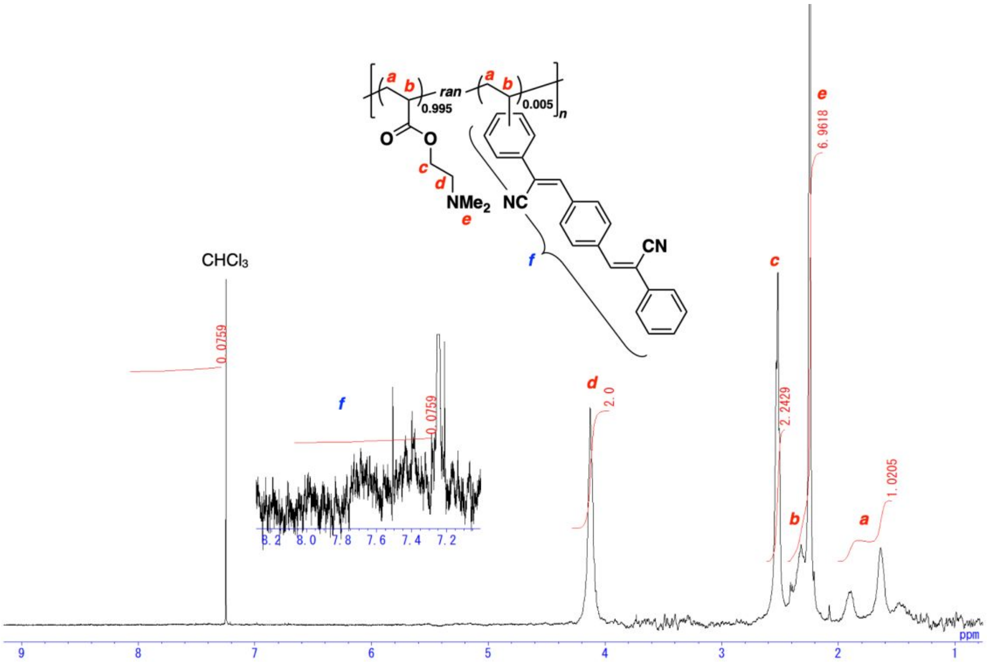

Figure S11. ^{1}H NMR spectrum of **3** in $CDCl_3$.

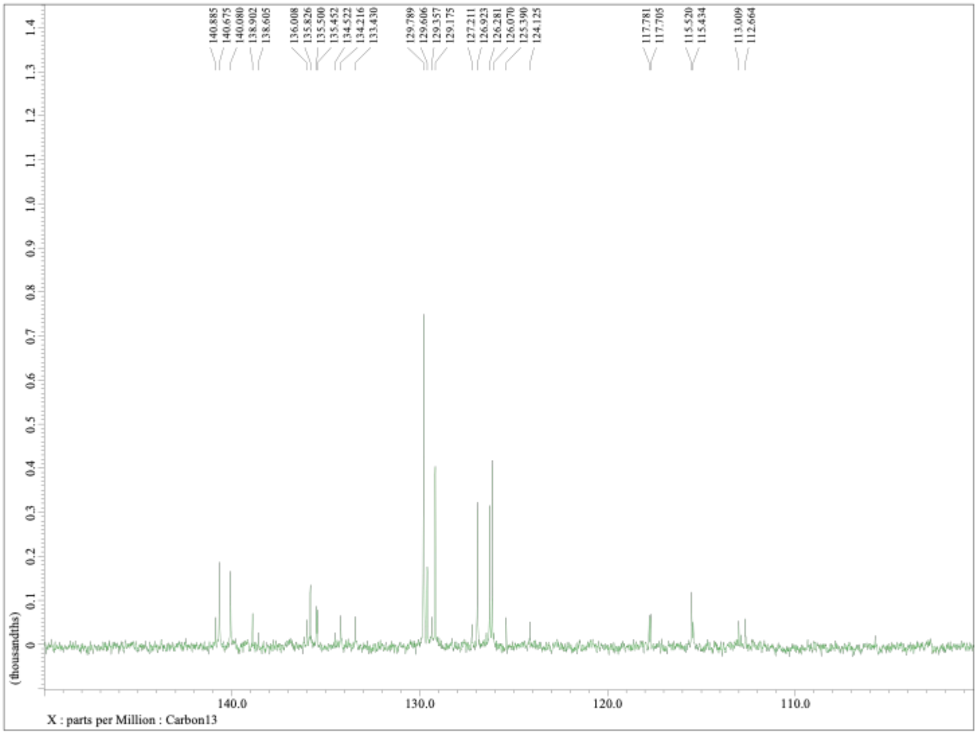

Figure S12. ^{13}C NMR spectrum of **3** in $CDCl_3$.

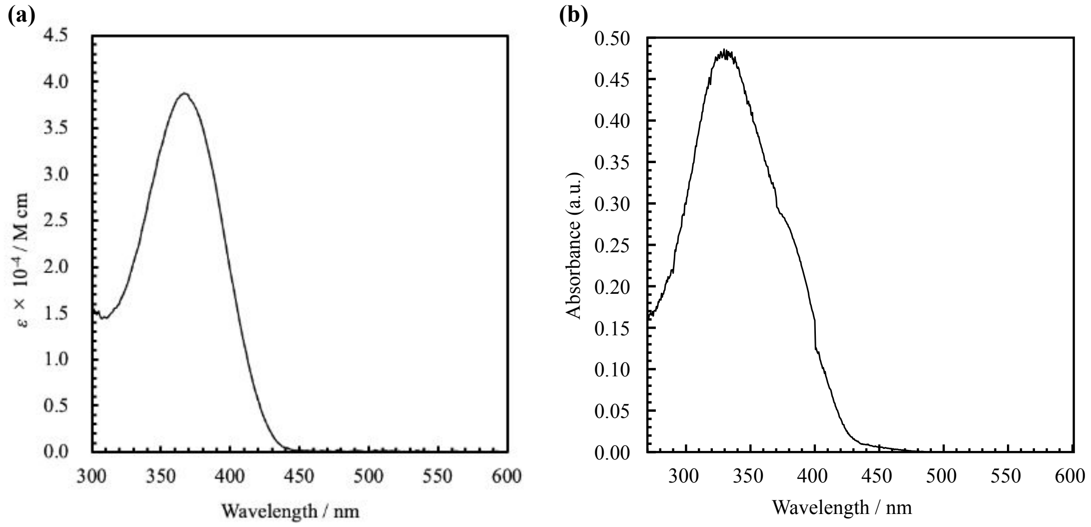

Figure S13. Absorption spectra of (a) **3** in CH_2Cl_2 (2.8 μM) at 25 ˚C and (b) poly-CDSB$_{0.005}$ in CH_2Cl_2 (7.5 mg/5.0 mL) at 25 ˚C.

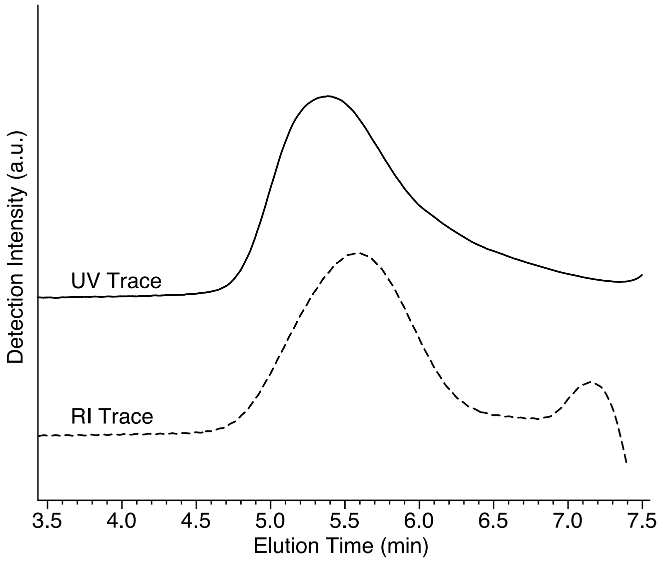

Figure S14. SEC trace of poly-CDSB$_{0.005}$ (eluent: DMF with 0.01 M of LiBr).

Reference

S1. Hayashi, S.; Hirai, R.; Yamamoto, S.; Koizumi, T. A Simple Route to Unsymmetric Cyano-substituted Oligo(p-phenylene-vinylene)s. *Chem. Lett.* **2018**, *47*, 1003.
